# Recurrent airway obstructions in a patient with benign tracheal stenosis and a silicone airway stent: a case report

**DOI:** 10.1186/1757-1626-1-226

**Published:** 2008-10-07

**Authors:** KB Sriram, PC Robinson

**Affiliations:** 1 Department of Thoracic Medicine, Royal Adelaide Hospital, Adelaide, South Australia 5000, Australia

## Abstract

Airway stents (silicone and metal stents) are used to treat patients with benign tracheal stenosis, who are symptomatic and in whom tracheal surgical reconstruction has failed or is not appropriate. However airway stents are often associated with complications such as migration, granuloma formation and mucous hypersecretion, which cause significant morbidity, especially in patients with benign tracheal stenosis and relatively normal life expectancy. We report a patient who had frequent critical airway obstructions over 8 years due to granuloma and mucus hypersecretion in a silicone airway stent. The problem was resolved when the silicone stent was removed and replaced with a covered self expanding metal stent.

## Background

Benign tracheal stenosis secondary to prolonged intubation and/or tracheostomy has been treated by airway stenting in selected patients1.

There are four categories of airway stents – silicone stents, balloon-dilated metal stents, self-expanding metal stents (SEMS) and covered SEMS. There are no randomized controlled trials comparing different stents and each category has its merits and limitations [1]. The silicone Dumon^® ^stent (Novatech, Boston Medical, Massachusetts, USA) is widely popular and often used to treat benign tracheal stenosis. Complications include stent migration, granuloma formation and mucostatsis [2]. Covered SEMS, such as the Ultraflex™ stent (Boston Scientific, Massachusetts, USA) are more biocompatible and some reports suggest fewer complication rates compared with silicone stents. We report a patient who developed major complications with a Dumon^® ^stent which were ameliorated when it was replaced by an Ultraflex™ stent.

## Case report

A 19 year old woman developed Guillain-Barre syndrome and respiratory failure in 1999, whilst living in a different city. Prolonged intubation followed by tracheostomy was complicated by tracheal stenosis. Tracheal reconstruction surgery was performed but was unsuccessful in maintaining airway patency. Immediately afterwards a 12 mm × 40 mm Dumon^® ^silicone stent was inserted. However this stent was complicated by frequent obstructions due to granulation tissue and mucous plugs.

Due to work commitments she relocated to our city in March 2006. In July 2006 she presented to the emergency department of our institution with acute dyspnea and stridor. Emergency bronchoscopy revealed extensive granulation tissue and mucous plugging within the Dumon^® ^stent. Over the next 16 months she had 9 emergency presentations with stent obstructions. Each episode was resolved with bronchoscopic piecemeal removal of granulation tissue and suctioning of mucus.

**Figure 1:**
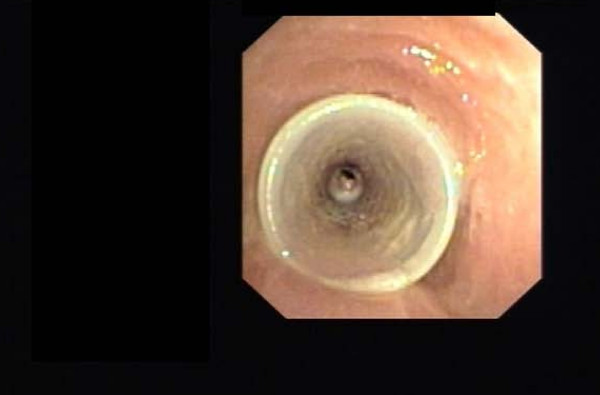
Silicone tracheal (Dumon^®^) stent has migrated proximally.

**Figure 2:**
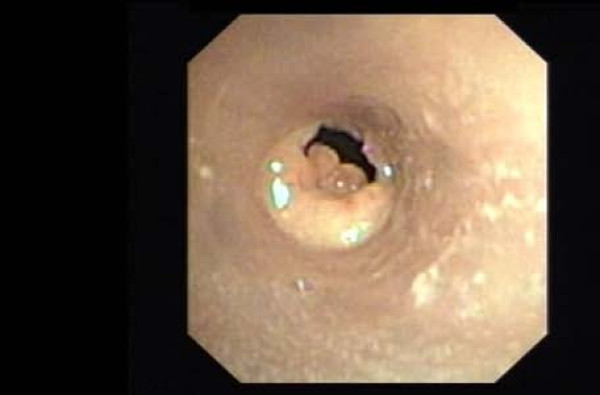
Granulation tissue obstructing distal end of silicone tracheal (Dumon^®^) Dumon stent.

In Nov 2007 she presented with severe respiratory distress and bronchoscopy revealed that the Dumon^® ^stent had moved proximally (Figure 1) with granulation tissue in the distal end (Figure 2) causing 90% obstruction in mid-trachea. The Dumon^® ^stent was removed and replaced with a 14 mm × 60 mm Ultraflex™ stent. The new stent was successful with resolution of tracheal obstruction. The patient has remained asymptomatic since, with no further episodes of airway obstruction.

## Discussion

Benign tracheal stenosis is most often caused by prolonged intubation and/or tracheostomy. 67–90% of patients who are intubated develop laryngotracheal injury and 12–14% develop tracheal stenosis [3]. Symptomatic benign tracheal stenosis is optimally treated by tracheal reconstruction surgery [4]. However, if this procedure is medically contraindicated or has failed previously then prosthetic stents are used to maintain airway patency.

Silicone stents are relatively inexpensive, generally do not fracture, resist extrinsic compression (e.g. from scar tissue or tumour) and can be easily repositioned or removed if required. However stent complications have been reported to occur in up to 42% of patients [5]. Long-term complications include migration (17%), granuloma (6%), and mucostasis causing airway obstruction (6%) [5]. One study found that 42% of patients require emergency bronchoscopy for stent related complications within 3 months of stent insertion [6]. Provocation of local inflammatory reaction results in growth of granulation tissue. Laser resection of in-stent granuloma must be performed with caution as there have been reports of flash fire and stent damage [4]. An additional problem with silicone stents is that the ratio of wall thickness to internal diameter is higher when compared to metal stents resulting in a smaller internal diameter in most cases [7]. Silicone stent-specific complications have raised the question of whether metallic stents might overcome some of these difficulties.

There has been increasing enthusiasm for using covered SEMS such as Ultraflex™ stents, which are built from a single layer of braided knitted flexible nitinol (nickel-titanium alloy) with an outer covering of silicone [1]. They have excellent flexibility, good biocompatibility and the self-expanding force is an important factor that enables the nitinol mesh to attach directly to the tracheal wall. While some reports have shown that there are few complications when SEMS are used to treat benign tracheal stenosis [8,9] others have found complications occurring in 52–87% of patients [3,4]. These include granuloma, mucus retention and stent fracture [3,4]. Such concerns have prompted the Food and Drug Administration to publish a cautionary advisory on the use of metallic stents in patients with benign airway disease [10].

Our report illustrates the complications associated with silicone airway stents when used to treat benign tracheal stenosis. Interestingly while the patient had frequent airway obstructions with the Dumon^® ^stent, she did not experience these with the Ultraflex™ stent. Careful patient selection and recognition of complications is paramount before considering patients with benign tracheal stenosis for treatment with prosthetic airway stents.

## Consent

Written informed consent was obtained from the patient for publication of this case report and accompanying images. A copy of the written consent is available for review by the Editor-in-Chief of this journal.

## Competing interests

The authors declare that they have no competing interests.

## Authors' contributions

KBS reviewed the case notes and prepared the manuscript. PCR read and approved the final manuscript.
